# Evolution of Coronary Stent Platforms: A Brief Overview of Currently Used Drug-Eluting Stents

**DOI:** 10.3390/jcm12216711

**Published:** 2023-10-24

**Authors:** Pierre Brami, Quentin Fischer, Vincent Pham, Gabriel Seret, Olivier Varenne, Fabien Picard

**Affiliations:** 1 Department of Cardiology, Cochin Hospital, Hôpitaux Universitaire Paris Centre, Assistance Publique des Hôpitaux de Paris, 75014 Paris, France; pierre.brami@aphp.fr (P.B.); quentin.fischer@aphp.fr (Q.F.); pham.qtv@gmail.com (V.P.); gabriel.seret.cardio@gmail.com (G.S.); olivier.varenne@aphp.fr (O.V.); 2 Département Santé, Université Paris-Cité, 75006 Paris, France; 3 INSERM U970, Paris Cardiovascular Research Center (PARCC), European Georges Pompidou Hospital, 75015 Paris, France

**Keywords:** coronary stents, percutaneous coronary intervention, drug-eluting stent, coronary heart disease

## Abstract

Cardiovascular disease, including ischemic heart disease, is the leading cause of death worldwide, and percutaneous coronary interventions (PCIs) have been demonstrated to improve the prognosis of these patients on top of optimal medical therapy. PCIs have evolved from plain old balloon angioplasty to coronary stent implantation at the end of the last century. There has been a constant technical and scientific improvement in stent technology from bare metal stents to the era of drug-eluting stents (DESs) to overcome clinical challenges such as target lesion failure related to in-stent restenosis or stent thrombosis. A better understanding of the underlying mechanisms of these adverse events has led DESs to evolve from first-generation DESs to thinner and ultrathin third-generation DESs with improved polymer biocompatibility that seems to have reached a peak in efficiency. This review aims to provide a brief historical overview of the evolution of coronary DES platforms and an update on clinical studies and major characteristics of the most currently used DESs.

## 1. Introduction

For the last 60 years, myocardial revascularization has benefited from the emergence and development of interventional cardiology to establish percutaneous coronary intervention (PCI) as the leading intervention for ischemic heart disease over coronary artery bypass grafting (CABG). At the beginning of PCI history, Sven Seldinger first developed in 1953 a safe and reproductive transcutaneous arterial puncture technique, while Mason Sones was the first physician to perform a coronary angiography involuntarily in 1958 during an aortography [1,2]. Andreas Grüntzig was the pioneer of PCI with the first-ever coronary balloon angioplasty in humans on 16 September 1977 in Zurich [3]. He used a double-lumen balloon catheter (one lumen dedicated to pressure monitoring) to treat a proximal left anterior descending artery stenosis in a 38-year-old patient and led the way to the so-called plain old balloon angioplasty (POBA). 

Several evolutions of the balloon angioplasty catheter were made in order to navigate more easily and to achieve better crossing of the lesion. Refinements of the technique led to the over-the-wire catheters and then the monorail system designed by Tassilo Bonzel and Paul Yock [4]. Nevertheless, despite the initial enthusiasm, POBA came with several complications, such as acute vessel closure related to traumatic vessel dissection (8–15% of cases) and early elastic recoil (5–10% of cases), requiring emergency repeat dilatation or bailout CABG. Beyond the intervention period, neointimal proliferation and negative remodeling secondary to vessel trauma were responsible for a 20–30% rate of restenosis at 6 months, hence requiring new angioplasty interventions [5,6] or bypass surgery.

**Figure 1:**
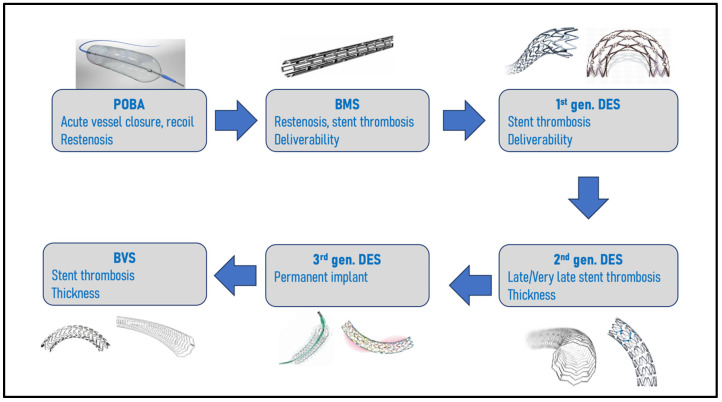
Stent platform evolution to overcome challenges.

Stents were then developed to overcome the acute complications of POBA, and Jacques Puel was the first to perform angioplasty with a bare-metal stent (BMS) in humans in March 1986 in Toulouse (France). PCI with BMS implantation (the Palmaz-Schatz model) showed its superiority against POBA in two important trials [7,8]. Although the rate of abrupt vessel closure considerably decreased, the main limitations were then acute and subacute stent thrombosis (ST), with rates as high as 20% despite anticoagulation therapies. This dreadful complication was attenuated by the introduction of a double antiplatelet regimen (ticlopidine and aspirin) instead of anticoagulation after BMS implantation [9]. However, because stents promote barotraumatism to the coronary artery medial layer, in-stent restenosis (ISR) remained a major issue, with clinically significant rates of ISR estimated at 20–30% at 6–9 months [6]. Many designs, expansion mechanisms, and coatings, such as gold plating or adjunctive therapies, were tested in an attempt to overcome this complication until the development of drug-eluting-stents (DESs), which would use the stent scaffold as a vehicle for local antiproliferative, anti-inflammatory and anti-restenosis drug. Figure 1 describes the key evolutions to overcome PCI pitfalls. This review aims to describe the initial generations of DESs and the principal clinical studies and characteristics of the latest generation of most used DESs.

## 2. First and Second-Generation DESs

The Cypher™ (Cordis, Santa Clara, CA, USA) was the first DES to receive the CE mark in 2002 and FDA approval in 2003. It was made of a stainless steel platform (strut thickness 132 µm) coated with polyethylene-co-vinyl acetate/poly n-butyl methacrylate as a polymer for sirolimus elution. The Cypher™ demonstrated its superiority against BMS to reduce ISR and target lesion revascularization (TLR) in de novo lesions in the two randomized trials, RAVEL and SIRIUS. In RAVEL, major cardiovascular adverse events (MACE) at 1 year decreased from 28.8% to 5.8% (*p* < 0.001), and in SIRIUS MACE at 270 days decreased from 18.9% to 7.1% (*p* < 0.001) largely because of a reduction in TLR [10,11]. The Taxus™ DES (Boston Scientific, Marlborough, MA, USA) received the CE mark in 2003 and then was FDA-approved in 2004. It was made of stainless steel (strut thickness 140 µm), but the polystyrene-block-isobutylene-block-styrene polymer eluted paclitaxel, a cytostatic antineoplastic drug. It also demonstrated its superiority at 9 months against BMS for de novo lesion in the TAXUS I trial regarding ISR (decrease from 39.6% to 15.4%, *p* < 0.01) and TLR (decrease from 19.0% to 9.9%, *p* = 0.001) [12]. When the two first-generation DESs were compared against each other, no statistical difference was found at 8 months in the rate of MACE or restenosis for de novo lesion in the REALITY trial, although the in-stent late loss was more important for the Taxus™ stents [13]. For ISR lesions, the Cypher™ seemed to have better outcomes regarding restenosis and TLR at 1 year in the secondary analysis of the ISAR DESIRE trial [14]. In addition to deliverability issues and angiographic visibility, long-term data started to alert concerning a high rate of late and very late ST [15,16]. At the 2006 European Society of Cardiology congress, many concerns were raised after the presentation of results showing an increase in total death and Q-wave MI in the DES compared with BMS [5]. They were linked to chronic inflammation, delayed strut-re endothelialization, and arterial healing [17]. This led to the development of the second generation of DESs.

The second generation of DESs was the result of a refinement work of several components. First, as DES platforms needed more deliverability, flexibility, and resistance to fracture, stainless steel was abandoned for cobalt–chromium and platinum–chromium alloys. These metal alloys are permitted to produce thinner struts (80–90 µm) compared to the thick stainless steel struts used so far (120–130 µm). This reduced thickness was supported by the ISAR-STEREO trial, which found a significant reduction within one year of angiographic restenosis (15.0% vs. 25.9%, *p* = 0.003) and of restenosis-driven clinical events (8.6% vs. 13.8%, *p* = 0.005) between patients treated with thin-struts (50 µ) or thick-struts (100 µm) BMS [18]. Those better clinical results also demonstrated in the ISAR-STEREO 2 trial [19], could be explained by a reduced contact surface of the body cells to the vessel, which attenuates the vascular injury responsible for inflammation and thrombosis, allowing faster re-endothelialization and decreased neointimal proliferation [20]. More recently, a meta-analysis confirmed the association between strut thickness and the occurrence of ST and MI [21]. Second, some stents used derivatives of the previously employed sirolimus. Everolimus and Zotarolimus are semi-synthetic agents that have the same mechanism of action as sirolimus but with enhanced lipophilic properties, which could result in better biodisponibility [22,23]. Finally, due to the late ST occurring with the first generation of DESs, new, more biocompatible polymers were developed, enabling faster drug elution and providing better endothelial coverage [24,25]. 

The first second-generation DES on the market in 2004 was the Endeavor™ (Medtronic, Minneapolis, MN, USA), with thinner struts (91 µm) of cobalt–chromium alloy eluting zotarolimus. It demonstrated better clinical outcomes at 5 years compared to the Cypher™ sirolimus-eluting stent (SES) with reduced MACE (14% vs. 22.2%, *p* = 0.05) driven by significantly less cardiac death/myocardial infarction (MI) (1.3% vs. 6.5%, *p* = 0.009) in the ENDEAVOR III study [26]. Later, in 2007, Medtronic changed its polymer for the dedicated company-designed polymer Biolynx™ and renamed it Resolute™. The Xience™ V (Abbott Vascular, Abbott Park, IL, USA) was available in 2008; it was made of a cobalt–chromium alloy with thin struts (81 µm) eluting everolimus from a double-layer polymer. It demonstrated better clinical outcomes at 5 years compared to the Taxus™ paclitaxel-eluting stent (PES), with reduced cardiac death (1.5% vs. 7.3%, *p* = 0.015) and ischemia-driven MACE (8.0% vs. 18.1%, *p* = 0.018) [27]. The Promus Element™ launched in 2012 (Boston Scientific, Marlborough, MA, USA) has a platinum–chromium alloy platform with thin struts (81 µm) and a durable polymer (DP) eluting everolimus similar to the Xience™ DES. The PLATINUM trial demonstrated its non-inferiority compared to the Xience™ for TLF at 5 years (Promus™ 9.1% vs. Xience™ 9.3%, *p* = 0.87) [28]. 

All the data accumulated on second-generation DESs suggested that these new platforms could be used with a shorter duration of double antiplatelet therapy (DAPT) of 3 to 6 months in the setting of elective PCI in comparison to the 12 months that was recommended with first-generation DESs to avoid bleeding complications without the expense of increased ischemic risk [29]. 

## 3. Third Generation DESs

Although long-term efficacy and safety outcomes were better than BMSs and first-generation DESs, concerns about late and very late ST persisted due to hypersensitivity reaction, residual inflammation, and delayed healing at later stages associated with DESs with permanent polymers [30]. Based upon these hypotheses, manufacturers developed a new generation of DES with biodegradable polymers (BP) or without polymer (polymer-free) drug-coated stents (DCS). They also kept improving deliverability and conformability to achieve higher procedural success in complex lesions (calcifications, bifurcations, left-main). Therefore, the latest generation of DESs differ not only by the eluted drug but also by the presence/absence of polymer, the type of polymer (permanent/degradable), the metallic alloy, the design and the number of connectors, and the strut thickness. 

**Figure 2:**
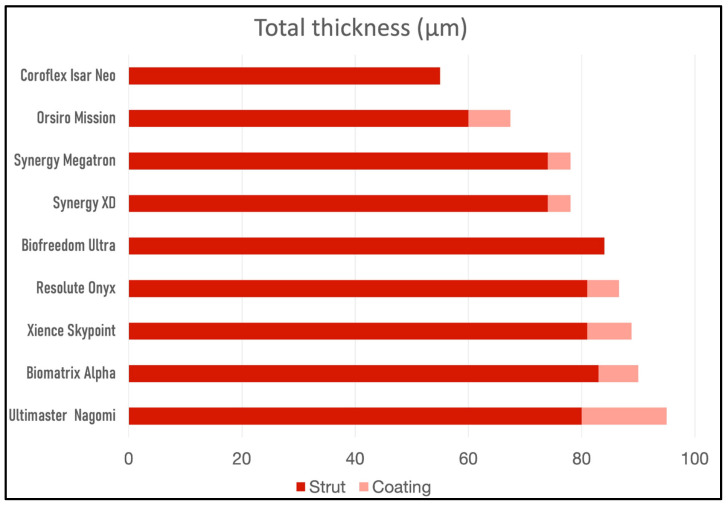
Stent thickness.

Their characteristics are summarized in Table 1. Figure 2 shows a comparison between them according to their total thickness.

### 3.1. Durable Polymer DESs

#### 3.1.1. Xience Skypoint™ (Abbott Vascular) 

**Figure 3:**
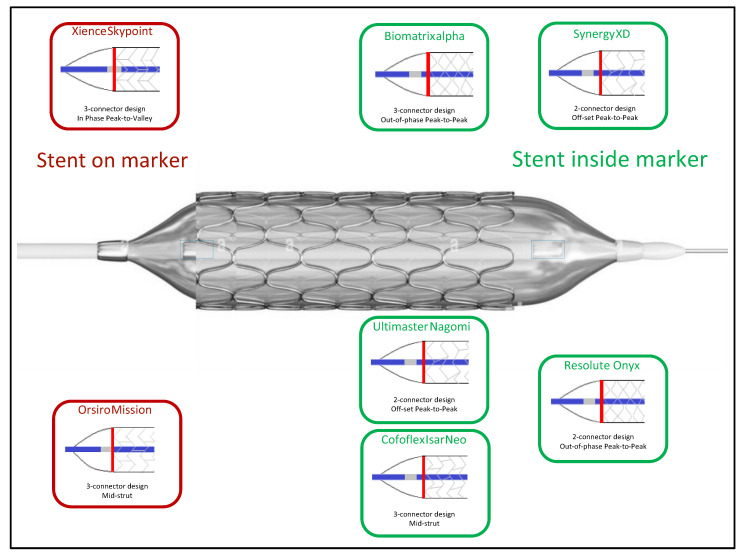
Stent structure and distance from balloon marker.

The Xience Skypoint™ is a cobalt–chromium alloy with a strut thickness of 81 µm (for all sizes), covered with a non-erodible, durable polymer matrix based on poly(n-butyl methacrylate) and a copolymer of vinylidene fluoride and hexafluoropropylene). The matrix elutes everolimus at a concentration of 100 μg/cm^2^, released gradually (80% at 30 days and 100% at 120 days). The stent alloy is built on the Multi-Link™ platform with three links per ring that have better longitudinal strength and avoid compression or deformation. There are two radiopaque mid-markers on the balloon at each end. Figure 3 shows the differences between DES platforms according to the location of the markers. It is the last iteration of the Xience™ line-up (Xience V™, Xience Prime™, Xience Xpedition™, Xience Alpine™, Xience Sierra™). It was first evaluated in the SPIRIT II trial, a single-blind randomized trial evaluating the Xience V™ everolimus-eluting stent (EES) against the Taxus™ PES in 300 patients. The late loss was lower in the Xience™ EES compared to the Taxus™ PES (0.11 ± 0.27 vs. 0.36 ± 0.39; *p* < 0.0001). The incidence of MACE was low and comparable between both treatment arms [31]. The five-year clinical follow-up results found lower cardiac mortality and ischemia-driven MACE in the Xience™ EES group (1.5% vs. 7.3% and 8.0% vs. 18.1%; *p* = 0.015 and *p* = 0.018, respectively). In addition, ST was lower in the Xience™ EES group (0.9% vs. 2.8%). No definite ST events were observed after two years in the Xience™ EES arm [27]. The SORT OUT IV trial compared Cypher™ (Cordis) SES to the Xience V™ and Promus Element™ EES 2774 in patients. At 9 months, the composite primary endpoint (cardiac death, MI, definite ST, target vessel revascularisation) occurred in 4.9% in the Xience™/Promus™ EES group and 5.2% in the Cypher™ SES group, establishing the non-inferiority of the EES (*p* = 0.02 for non-inferiority). The results were sustained at 18 months [32]. The BIOFLOW IV trial randomized 575 patients to receive Xience Prime™ EES or Orsiro^®^ (Biotronik, Berlin, Germany) SES. At 12 months, the primary endpoint TVF (cardiac death, target vessel Q-wave or non-Q-wave MI, CABG or clinically-driven target vessel revascularisation) occurred in 5.5% of the Xience™ EES group and 6.6% of the Orsiro^®^ SES group (*p* < 0.001 for non-inferiority) [33]. Finally, the XIENCE SHORT DAPT program consisted of three prospective, single-arm studies exploring two different DAPT durations (1 month or 3 months) in patients undergoing PCI with Xience™ EES. The primary objective was to evaluate the safety of a short duration of DAPT (3 months in XIENCE 90 and 1 month in XIENCE 28) in 3652 high-bleeding risk patients undergoing PCI with the Xience™ EES, with the exclusion of left main coronary lesions, restenotic lesions of a previously stented segment, chronic total occlusion or lesions treated with overlapping stents. The primary endpoint of all-cause mortality or MI was similar between the two groups (7.3% vs. 7.5%; *p* = 0.41). The key secondary endpoint of Bleeding Academic Research Consortium type 2-5 bleeding was lower with 1-month DAPT compared with 3-month DAPT (7.6% vs. 10.0%; *p* = 0.012). However, major bleedings (BARC 3-5) did not differ at 12 months (3.6% vs. 4.7%; *p* = 0.082) but was lower with 1-month DAPT at 90 days (1.0% vs. 2.1%; *p* = 0.015) [34]. These results support the safety of a short DAPT in high-bleeding risk patients treated with Xience™ EES.

#### 3.1.2. Resolute Onyx™ (Medtronic)

The Resolute Onyx™ is a thin strut (81 µm) cobalt–chromium alloy shell stent with a platinum–iridium core for enhanced radio-opacity, longitudinal stent stability, and high radial strength. It is a zotarolimus-eluting stent (ZES), which is a sirolimus-derived antiproliferative agent with more lipophilic properties, allowing better and faster penetration of the arterial wall. The Resolute Onyx™ is based on the previous generation of Endeavor Resolute™ ZES (Medtronic) but with a new BioLinx™ polymer that facilitates drug release (85% during the first 60 days) and a redesigned platform (single sinusoidal formed wire with local laser fusion). The struts are circumferentially covered by a 5.6 µm thick coating. The Resolute Onyx™ is an evolution of the Resolute Integrity™ with thinner struts (90 µm for the Integrity), better radio-opacity of the platform, and the markers combined with a refined catheter delivery system. It was first evaluated in the DUTCH PEER trial, a single-blinded randomized trial evaluating the non-inferiority of the Resolute Integrity™ ZES against the Promus Element™ EES in an all-comers population of 1810 patients and 2371 lesions. At 12 months, there was no difference between the Resolute™ ZES and Promus™ EES groups for TLF (6% vs. 5%; HR 1.17 95% CI 0.80–1.71; *p* = 0.42 for difference, *p* = 0.006 for non-inferiority). The definite ST remained rare without any difference (<1% vs. <1%; HR 0.50 95% CI 0.13–2.00; *p* = 0.51) [35]. The TWENTE trial was a patient-blinded non-inferiority randomized trial to assess the performance of the Resolute Integrity™ ZES against the Xience™ (Abbott Vascular) EES in 1391 unselected "real-world" patients with the exception of ST-elevation myocardial infarction (STEMI), with a significant proportion of complex lesions (70% of class B2 or C). At 12 months, there was no difference between the Resolute™ ZES and the Xience™ EES groups for TVF (composite of cardiac death, target-vessel-related MI, or clinically driven target vessel revascularisation) (8.2% vs. 8.1%, *p* = 0.94). Probable or definite ST was infrequent in both groups (1.0% vs. 0.9%, *p* = 0.59) [36]. The SORTOUT VI trial was an open-label, randomized, non-inferiority trial evaluating the Resolute Integrity™ ZES against the Biomatrix (Biosensors International, Singapore, Singapore) Biolimus A9-eluting stent (BES) with BP in 2999 patients presenting with stable coronary artery disease or acute coronary syndrome (ACS). At 12 months, the primary endpoint (composite of cardiac death, MI not attributable to non-target revascularization and TLR) occurred in 5.3% in the Resolute™ ZES group and 5.0% in the Biomatrix BES group, meeting the non-inferiority prespecified criteria (*p* = 0.004) [37]. To overcome the difficulty of assessing low-frequency and long-term cardiovascular events in a reliable manner, the GLOBAL RESOLUTE Trial Program pooled the results from ten prospective clinical trials across the world evaluating the Resolute™ ZES with identical adverse event definition and adjudication. The cumulative incidence at 5 years was 13.4% for TLF, 5.0% for cardiac death, 4.4% for target-vessel-MI, and 6.3% for target vessel revascularization. The probable or definite ST incidence at 5 years was 1.2%, comprising 0.5% early ST (<30 days). These results confirmed the low rate of cardiac events following treatment with Resolute™ ZES [38]. The RESOLUTE ONYX CORE trial was an open-label, non-inferiority study comparing the Resolute Onyx™ ZES against the Resolute Integrity™ ZES using the historical cohort from the RESOLUTE-US trial matched with a propensity score as a control arm in 75 patients. At 8 months, the in-stent late lumen loss was 0.24 ± 0.39 for the Resolute Onyx™ ZES group and 0.36 ± 0.52 for the Resolute Integrity™ ZES group, meeting the non-inferiority (*p* < 0.001) and the prespecified criteria for superiority (*p* = 0.029). Secondary endpoints evaluated clinical outcomes at 8 months with 6.7% of TLF and 4% clinically driven TLR in the Resolute Onyx™ ZES group compared to 11.2% and 8.2%, respectively, in the Resolute Integrity™ ZES group. These results may be explained by the thinner struts and modified design with a swaged shape that affects endothelialization, neointimal hyperplasia, and endothelial shear stress [39]. The ONYX ONE trial investigated a strategy of short DAPT (1 month) in a high bleeding risk population, randomized to be treated with DP Resolute Onyx™ ZES or the polymer-free Biofreedom™ BES. Patients had a clinical indication for PCI and were considered a high bleeding risk. They were randomized to receive 1 month of thrombosis prophylaxis (DAPT or single antiplatelet plus an oral anticoagulant agent). More than 50% were treated for an ACS. At 1 year, the primary outcome composed of cardiac death, MI, or ST occurred in 17.1% of the Resolute™ ZES group and 16.9% of the Biofreedom™ BES group meeting the non-inferiority (*p* = 0.01). The secondary outcome TLF occurred in 17.6% of the Resolute™ ZES group and 17.4% of the Biofreedom™ BES group (*p* = 0.007 for non-inferiority) [40]. Finally, the BIORESORT trial was a three-arm, randomized, assessor-patient-blinded trial evaluating the safety and efficacy of the DP Resolute™ ZES against the BP Orsiro^®^ SES and Synergy™ EES in an all-comer population of 3514 patients. 70% had ACS, among whom 30% had STEMI. At 1 year, the primary composite endpoint TVF (cardiac death, vessel-related MI and clinically driven target vessel revascularisation) was met by 5% of the Synergy™ EES group, 5% of the Orsiro^®^ SES group and 5% of the Resolute™ ZES group; Resolute™ ZES met the non-inferiority prespecified criteria against Orsiro^®^ SES (*p* < 0.0001) and Synergy™ EES (*p* < 0.0001). The 1-year rate of definite or probable ST was similar among the treatment groups (<1%). It is to be noted that the potential benefits of BP stents over DP stents might be only perceived after one year [41]. 

### 3.2. Biodegradable Polymer DESs

#### 3.2.1. Synergy™ XD (Boston Scientific)

The Synergy™ is a thin-strut (74–81 µm) platinum–chromium metal alloy platform eluting everolimus (100 µg/cm^2^) from an ultrathin coating (4 µm) of bioabsorbable poly(D,L-lactide-coglycolide) polymer applied only to the abluminal surface. Everolimus is eluted within 3 months, and the polymer absorption is complete within 4 months, which could facilitate stent endothelization and allow shorter DAPT duration. Its last iteration is the Synergy™ XD, which differs from the Synergy™ only in the catheter characteristics. The company also developed the Synergy™ Megatron for large vessels (mostly left-main trunk); it has marginally thicker struts (89 µm) and is designed with a higher number of peaks and connectors, allowing it to expand until 6.0 mm without losing its radial strength. It was first evaluated in the randomized EVOLVE trial, which compared the Synergy™ stents with two doses of everolimus against the Promus Element™ (Boston Scientific) DP EES in 291 patients. One formula had a dosage of everolimus similar to the Promus™; the other one had half the dose. There was no difference in terms of TLF, a composite of cardiac death, MI related to the target vessel, or ischemia-driven revascularization, between the two Synergy™ EES groups (Synergy and Synergy half-dose) against the Promus™ EES group at 30-days (1.1% vs. 0%; *p* = 0.49 and 3.1% vs. 0%; *p* = 0.25 respectively) and 6 months (2.2% vs. 3.1%; *p* = 1.00 and 4.1% vs. 3.1%; *p* = 0.72 respectively) [42]. The 5 years results of the EVOLVE trial found no significant differences in the rates of TLF or individual components between groups. There was a trend to lower TLR in the Synergy™ EES group against the Promus Element™, and no ST (definite or probable) occurred in any groups [28]. Following this first trial, the EVOLVE 2 trial evaluated the clinical efficacy and safety of the Synergy™ EES for regulatory approval in a broad population against the Promus Element™ (Boston Scientific) EES in 1684 patients. Of note, patients with TIMI 0 flow, left main coronary artery lesion, chronic total occlusion, and recent STEMI were excluded from the trial. At 12 months, the Synergy™ EES was not inferior to the Promus Element™ EES regarding the primary endpoint of TLF (6.7% vs. 6.5%; *p* < 0.025). There was also no difference for ST at 12 months (0.4% vs. 0.6%; *p* = 0.5) [43]. The 4-year results of the EVOLVE II trial supported the long-term safety and efficacy of the Synergy™ EES who remained non-inferior to the Promus Element™ EES regarding TLF (12.7% vs. 12.3%; *p* = 0.83) with a low rate of ST (0.6% vs. 0.9%; *p* = 0.54) [44]. Further, the EVOLVE SHORT DAPT trial evaluated a short duration of DAPT (3 months of aspirin + P2Y12) in patients treated with Synergy™ EES against a propensity-adjusted historical 12-months DAPT cohort derived from 3 studies including various limus eluting stents. Patients were enrolled if they met at least one criterion for high bleeding risk. Patients with recent MI, ISR, chronic total occlusion, and left main lesion were excluded. The analysis of the period ranging from 3 to 15 months post-PCI regarding the incidence of death/MI between the two groups demonstrated its non-inferiority (5.6% vs. 5.7%; *p* = 0.01). 3-months DAPT was also non-inferior regarding ST (0.2% vs. 0.63%; *p* = 0.01). Surprisingly, the rate of bleeding events was not different between groups (6.3% vs. 4.2%; *p* = 0.98), potentially because of the analytic methodology using worst-case scenario analysis in the control group when the timing of occurrence of a bleeding event was not known which could have induced a bias against the test group [45]. Finally, in the Swedish Nationwide SCAAR registry, which described the clinical outcomes of the Synergy™ EES against other new-generation DES in a large real-life unselected population matched with a propensity score, the cumulative incidence of ISR, ST, death, and MI was not different between the Synergy™ EES and the other-DES groups. These results support the safety and efficacy of the Synergy™ EES in a real-life population [46].

#### 3.2.2. Orsiro^®^ Mission (Biotronik)

The Orsiro^®^ stent is a cobalt–chromium stent platform designed in a double-helix structure. The metal alloy allows for ultrathin struts (60–80 µm), which provide greater flexibility for better stent delivery. It has a hybrid coating consisting of active and passive components to improve biocompatibility. The outer layer contains a BIOlute^®^ active coating made from biodegradable poly-L-lactide that slowly degrades over 12 to 15 months and releases sirolimus at a dose of 1.4 µg/mm^2^ over a 3-month period. The Orsiro^®^ Mission is an evolution of the Orsiro^®^ stent, only incorporating mild catheter modifications. The BIOlute^®^ active coating has an abluminal thickness of 7.5 µm and a luminal thickness of 3.5 µm. This asymmetric circumferential distribution ensures adherence of the polymer to the stent platform in regions of increased stress during stent expansion. The inner layer contains proBIO^®^ passive coating, made of 80 nm of silicon carbide that eliminates the interaction between the metal alloy and surrounding tissue, which could reduce thrombogenicity and promote endothelialization. It was first evaluated in the BIOSCIENCE trial, which randomized 3139 patients between Orsiro^®^ SES or Xience™ (Abbott Vascular) EES. At 12 months, the Orsiro^®^ SES was non-inferior to the Xience™ EES regarding the primary endpoint of TLF (cardiac death, target-vessel MI and clinically-indicated TLR) (6.7% vs. 6.7%; *p* = 0.0004 for non-inferiority). There was no difference in the rate of definite or probable ST among the two groups (2.8% vs. 3.4%; *p* = 0.45). In the subgroup analysis of patients presenting with STEMI, the Orsiro^®^ SES was associated with a lower incidence of TLF (3.3% vs. 8.7%) [47]. The BIOFLOW V was a randomized superiority trial comparing the Orsiro^®^ SES to the Xience™ EES in 1334 patients. At 12 months, the rate of the primary endpoint of TLF occurred significantly more in the Xience™ EES group than in the Orsiro^®^ SES group (10% vs. 6.0%; *p* = 0.04), a result mainly driven by a higher rate of target-vessel MI (8% vs. 5%; *p* = 0.015). The rate of definite or probable ST was low in both groups in this trial (<1%) [48]. Nevertheless, the 5-year results of the trial found similar rates of TLF among the two groups (15.5% vs. 12.3%; *p* = 0.108), although a lower incidence of target-vessel MI and late/very late ST was noted in patients treated with Orsiro^®^ SES (6.6% vs. 10.3%; *p* = 0.015 and 0.3% vs. 1.6%, *p* = 0.021, respectively [49]. The BIOSTEMI trial, therefore, randomized patients with STEMI undergoing primary PCI to receive Orsiro^®^ SES or Xience™ EES. At 12 months, the primary endpoint of TLF occurred in 4% of patients treated with Orsiro^®^ SES and 6% of patients treated with Xience™ EES. The prespecified criterion for superiority of the Orsiro^®^ SES compared to the Xience™ EES was met (difference –1.6 percentage points; rate ratio 0.59, 95% Bayesian credibility interval 0.37–0.94; posterior probability of superiority 0.986). This difference was driven by fewer ischemia-driven TLRs [50]. The 2-year results of the BIOSTEMI trial found consistent results with a persisting superiority of the Orsiro^®^ SES compared to the Xience™ EES regarding TLF. There were no significant differences in rates of cardiac death, target-vessel myocardial reinfarction, or definite ST [51]. Finally, the BIONYX trial, which was a trial evaluating the Orsiro^®^ SES against the Resolute Onyx™ (Medtronic) ZES in 2488 patients eligible for PCI, found that the primary composite endpoint of TVF (cardiac death, target-vessel related MI or clinically indicated target vessel revascularisation) was met in 4.5% of the Resolute Onyx™ ZES group and 4.7% of the Orsiro^®^ SES group, establishing non-inferiority between the two stents (*p* = 0.0005). Definite or probable ST occurred in 0.1% of the patients treated with Resolute Onyx™ ZES and in 0.7% of the patients treated with Orsiro^®^ SES [52]. 

#### 3.2.3. Biomatrix Alpha (Biosensors International)

The Biomatrix stents family are BESs. The first iterations (Biomatrix, Biomatrix Flex, Biomatrix Neoflex) were made of a stainless steel platform (strut thickness 120 µm), then evolved with the Biomatrix Alpha for the thin-strut platform (83 µm) made of cobalt–chromium and keeping all the other design elements. Biolimus is a semi-synthetic analog of sirolimus with a highly lipophilic profile for better absorption associated with a slower metabolism of its structure. The biolimus is applied at the abluminal surface at a concentration of 15.6 µg/mm^2^ in a polylactic acid biodegradable polymer (absorbed over 6 to 9 months). The Biomatrix BES was first evaluated in the LEADERS trial against the Cypher™ (Cordis) SES in 1707 patients in the setting of chronic or ACS. At 9 months, the primary endpoint (cardiac death, MI, or clinically driven target vessel revascularisation) occurred in 9% of patients treated with Biomatrix BES and 11% with Cypher™ SES, establishing non-inferiority (*p* = 0.003). The Biomatrix BES was also non-inferior for in-stent percentage diameter stenosis and ST [53]. The five-year results found a persisting non-inferiority between groups except for the subgroups of patients with STEMI and non-diabetics, for which patients treated with Biomatrix BES had better outcomes than those treated with Cypher™ SES. Very late definite ST (occurring after 1 year) was also significantly more frequent in the Cypher™ SES group than the Biomatrix BES group (2.2% vs. 0.6%; *p* = 0.003) with an overall incidence of 4.2% vs. 2.6% respectively [54]. The SORTOUT VIII trial further evaluated the Biomatrix Neoflex BES against the Synergy™ EES in 1369 patients. At 12 months, 4% of patients treated with Synergy™ EES and 4.4% with Biomatrix BES met the primary composite endpoint of cardiac death, MI not clearly attributed to non-target lesion or clinically driven TLR meeting the criteria for non-inferiority (*p* < 0.001). Nevertheless, ST did not differ between the two groups (1.4% vs. 1.1%) [55], but device delivery failure was more frequent with Biomatrix BES compared to Synergy™ EES (3.0% vs. 1.8% respectively; *p* = 0.04). The BIODEGRADE trial compared the Biomatrix Flex BES against the Orsiro^®^ SES in 2341 patients. At 18 months, the primary endpoint of TVF (cardiac death, target vessel-related MI, or ischemia-driven TLR) occurred 2.9% in the Biomatrix BES group vs. 2.1% in the Orsiro^®^ SES group (*p* < 0.001 for non-inferiority). Only 0.2% of patients presented late definite ST [56]. Due to the higher rate of stent delivery failure with the stainless steel thick platform, the Biomatrix evolved into Biomatrix Alpha, a thinner cobalt–chromium (83 µm) platform. This new platform was evaluated in the BIOMATRIX ALPHA REGISTRY, a post-market surveillance registry with 400 patients, to evaluate the Biomatrix Alpha against its previous iterations. A propensity score with the historical LEADERS cohort was used for comparison, and it found that the incidence of MACE (cardiac death, MI, and clinically indicated TLR) at 9 months was similar (4.3% vs. 5.0%, HR 0.83; 95% CI 0.55–1.26; *p* = 0.38) [57]. 

#### 3.2.4. Ultimaster Nagomi™ (Terumo)

The Ultimaster Nagomi™ is an open-cell stent made of cobalt–chromium with a strut thickness of 80 µm. It incorporates a biodegradable polymer made of Poly(D,L-lactide-co-caprolactone) that gradually releases sirolimus (3.9 µg/mm stent length) in 3 to 4 months and simultaneously resorbs. The abluminal coating allows a gradient distribution in order to uniformly release the drug for effective neointimal hyperplasia inhibition. It was first evaluated in the CENTURY II trial, a randomized, controlled, non-inferiority trial comparing Ultimaster™ (first iteration) SES to Xience™ EES in 1101 patients. The primary composite endpoint, freedom from cardiac death, MI not clearly attributable to a non-target vessel and clinically driven TLR at five years, was 90.0% in the Ultimaster™ SES group and 91.1% in the Xience™ EES group (*p* = 0.54). Importantly, the rate of very late ST between one and five years was remarkably low at 0.2% in both study arms [58]. Following, the E-ULTIMASTER registry evaluated the performance of the Ultimaster™ SES in an all-comer population. The primary objective was to assess TLF (cardiac death, target-vessel MI, clinically driven TLR) at 1 year. A total of 37,198 patients were enrolled, and at 1-year follow-up, the occurrence of TLF was 3.2% across all patients [59]. More recently, the MASTER DAPT trial evaluated a 1-month DAPT against a longer course (6 months or 3 months for those who received oral anticoagulation) of DAPT in 4579 patients at high bleeding risk treated with the Ultimaster™ (first iteration or its evolution the Ultimaster Tansei™) SES. Three primary outcomes were reported: net adverse clinical events (death from any cause, MI, stroke, or major bleeding), MACE (death from any cause, MI, or stroke), and major or clinically relevant non-major bleeding. At 335 days, net adverse clinical events occurred in 7.5% of the abbreviated-therapy group and 7.7% of the standard-therapy group (*p* < 0.001 for non-inferiority). MACE occurred in 6.1% of the abbreviated-therapy group and 5.9% of the standard-therapy group (*p* = 0.001 for non-inferiority). However, major or clinically relevant bleedings were less frequent with short DAPT (6.5% vs. 9.4% in the standard therapy group; *p* < 0.001 for superiority). These results established the safety of 1-month DAPT after coronary stenting with Ultimaster™ DES in high bleeding risk patients without reducing its efficacy [60].

### 3.3. Polymer-Free Drug-Coated Stents

#### 3.3.1. Coroflex^®^ Isar Neo (B. Braun)

The Coroflex^®^ Isar Neo is a polymer-free SES designed with an ultrathin strut design (55 to 65 µm) made of cobalt–chromium. Compared to its last iteration (Coroflex^®^ Isar), the Coroflex^®^ Isar Neo was marginally thicker (+5 µm) and had a distal hydrophilic coating. The stent’s unique composition includes a sirolimus matrix coating on the abluminal surface, with a concentration of 1.2 µg/mm^2^. This sirolimus-based antiproliferative drug is combined with probucol, an excipient that controls the release of sirolimus over time (100% released over 90 days after implantation), providing effective drug delivery without polymer. The ISAR-TEST 5 trial is a randomized, controlled, non-inferiority trial that evaluated the Isar SES (which shares the same characteristics as the Coroflex^®^ Isar SES except for its stainless steel platform) against the Endeavor ZES in 3002 patients. At one year, the composite primary endpoint (cardiac death, MI related to target vessel, and TLR) occurred in 13.1% of the Isar SES group and 13.5% of the Endeavor ZES group (*p* = 0.006 for non-inferiority). The angiographic follow-up at 6–8 months showed similar angiographic restenosis rates (13.3% vs. 13.4% respectively; *p* = 0.95), and the probable/definite ST rates were not different between groups (1.1% vs. 1.2% respectively; *p* = 0.80) [61]. The 10-year outcomes of ISAR-TEST 5 found persistent results with no difference regarding the primary endpoint (43.8% vs. 43.0% respectively; *p* = 0.90). Probable/definite ST remained low and not different (1.6% vs. 1.9%, respectively; *p* = 0.58) [62]. More recently, the ISAR 2000 registry involved a large-scale international, single-armed, multicenter observational design, with 2877 patients treated with Coroflex^®^ Isar SES, of whom 1084 had ACS. At 9 months, the overall TLR rate was 2.3% for the entire patient population. Notably, there was no significant difference in the TLR rates between patients with ACS and those with stable coronary artery disease (2.6% vs. 2.1%, *p* = 0.389). These findings suggest that the Coroflex^®^ SES demonstrated safety and efficacy in routine clinical practice, with low rates of TLR and MACE in an unselected patient population [63]. 

#### 3.3.2. Biofreedom Ultra™ (Biosensors)

The Biofreedom Ultra™ is a polymer-free DCS made of cobalt–chromium thin struts (84–88 µm with a micro-structured abluminal surface delivering Biolimus A9). The transfer of the antiproliferative agent is complete within 28 days. It is an evolution of the Biofreedom™ (first iteration) that was made of stainless steel with thicker struts (112–120 µm). It was initially developed to respond to high bleeding risk patients who would benefit from short DAPT allowed by the absence of polymer that could facilitate rapid re-endothelialization and improve the healing process. It was first evaluated in the LEADERS FREE trial, a double-blinded randomized trial that compared the Biofreedom™ BES and the Gazelle BMS in 2432 high bleeding risk patients with a 1-month DAPT. At 390 days, the primary endpoint (composite of cardiac death, MI, or ST) occurred in 9.4% in the Biofreedom™ BES group and 12.9% in the Gazelle BMS group (*p* = 0.005 for superiority). Clinically driven TLRs were more frequent in the BMS group (5.1% vs. 9.8%, *p* < 0.001) [64]. It was then evaluated in the SORTOUT IX trial against the Orsiro^®^ (Biotronik) SES in 3151 patients with a high rate of ACS (52%) and complex lesions (60%). At 1 year, the composite primary endpoint of MACE (cardiac death, target-vessel-related MI, and TLR) occurred in 5.0% of the Biofreedom™ BES group and 3.7% of the Orsiro^®^ SES group; non-inferiority of the Biofreedom™ BES could not be demonstrated. There was a trend to a higher incidence of TLR with Biofreedom™ BES compared to Orsiro^®^ SES (3.5% vs. 1.3%) [65]. Finally, the BIOFREEDOM-QCA trial assessed the efficacy of the Biofreedom Ultra™ BES against its previous iteration, the Biofreedom™ BES, in 200 randomized patients at high bleeding risk treated with DAPT1 for 1 month. At 9 months, the Biofreedom Ultra™ BES was non-inferior to its predecessor for late-lumen-loss (0.34 ± 0.49 mm vs. 0.29 ± 0.37 mm, respectively; *p* = 0.005 for non-inferiority), and TLF was similar between groups (7.3% vs. 9.3%, respectively, *p* = 0.60) [66].

## 4. Leaving Nothing Behind

### 4.1. Bioabsorbable Vascular Scaffolds 

Bioabsorbable vascular scaffolds (BVSs) were supposed to represent the fourth revolution in interventional cardiology. The concept of their development that started two decades ago stands on the idea that stent scaffolds are only transiently necessary to the vessel within the first 6 months after their implantation to prevent acute recoil and maintain vessel integrity. After this period, the negative remodeling and neointimal formation provide a sufficient structure to the artery. Moreover, the degradation of stent scaffolds could allow restoration of the vasomotor and endothelial functions, reducing neoatherosclerosis and facilitating positive remodeling. The absence of long-term metal reduces the risk of stent thrombosis and leaves additional options for further revascularization, either surgically or percutaneously [67,68,69]. Several companies developed a BVS program; the two major ones were the Absorb (Abbott Vascular), made of poly-L-lactide (thickness 156 µm) eluting everolimus with a resorption time of 36 to 48 months and the Magmaris (Biotronik) made of magnesium (thickness 120 µm) first eluting paclitaxel and then sirolimus for its second iteration with a resorption time of 6 to 12 months [70,71,72]. Although the concept of BVS was a seducing idea that raised global enthusiasm in the scientific community, its development program encountered several concerns that led to its withdrawal from the market after the disappointing results of the ABSORB III trial that demonstrated higher TLF compared with the latest generation of DES driven by an increased risk of target-related MI and higher ST rates [73,74]. Many explanations to understand these deceptive results were explored: the importance of lesion preparation/vessel sizing/post dilatation performance ("PSP technique"), lesion selection with reference vessel size ≥2.50 mm, strut thickness and stent base composition (poly-L-lactide have been found to trigger thrombogenicity, inflammatory cell adhesion, and delayed endothelialization in porcine models). Despite these drawbacks, companies still run BVS development programs with the refinement of the devices [75].

### 4.2. The Future of DESs

Nanotechnology is another innovation that can enhance the performance of future DES. In the future, we should witness the design of a stent surface that can promote re-endothelialization and inhibit restenosis without compromising hemocompatibility. This engineering can be achieved by chemically and topographically modifying the nanosurface of the stent or depositing a thin film with various properties. The texturing of the surface at a nanoscale is known to regulate vascular cell adherence and proliferation by altering the biological response. Several nanoarchitectures are studied, such as nanotubes, nanoleaves, or nanopores. Titanium or titanium alloys seem to be well-suited to receive nanoscale modifications. Nanocoating of the stent surface with a biocompatible film can act as a barrier between the metal and the surrounding tissue and blood, improving vascular cell response. These coatings are made of polymers, biocompatible materials, or inorganics. Moreover, nanoparticles can be used to better control targeted-drug delivery with enhanced intracellular uptake, higher arterial wall concentration, and sustained drug release while reducing drug dosage for less toxicity and limiting off-axis effects [76]. 

Furthermore, integrated nanosensors in stents could provide real-time monitoring of lesion status, offering valuable insights into vascular healing. Sensors to detect endothelial coverage (related to the occurrence of late ST) or ISR are under development [77]. On another level, biomolecular vascular therapeutics are studied to prevent ISR. With a design similar to natural oligo-nucleotides or small-chain peptides, they offer high specificity and extensive customizability. They can have an effect on vascular gene editing, epigenetics, or biomimetic peptides. Advances in nanotechnology hold the promise of enhancing the precision and effectiveness of coronary stents, and these research initiatives open up new perspectives for improving long-term outcomes of coronary interventions [78,79].

## 5. Conclusions

Coronary stent platform evolution is a remarkable history of partnership between the industry engineering and the medical profession, leading major randomized clinical trials that have resulted in numerous devices that are highly efficient and safe. To date, with recent data, none of them seems to outperform the others due to excellent performances and refinements. Nevertheless, the saga of PCI keeps moving forward, and the future will bring new developments in the devices we use with BVSs or nanotechnologies in DESs.

## Figures and Tables

**Table 1:** Stent platform characteristics.

Stent Platform	Synergy XD™	Synergy Megatron™	Xience Skypoint™	Orsiro^®^ Mission	Resolute Onyx™	Biomatrix Alpha	Ultimaster Nagomi™	Coroflex^®^ Isar Neo	Biofreedom Ultra™
Metal alloy	platinum–chromium	platinum–chromium	cobalt–chromium	cobalt–chromium	cobalt–chromium	cobalt–chromium	cobalt–chromium	cobalt–chromium	cobalt–chromium
Active drug	everolimus	everolimus	everolimus	sirolimus	zotarolimus	biolimus A9	sirolimus	Sirolimus /probucol	biolimus A9
Strut thickness	Ø 2.25–2.75: 74 µm Ø 3.0–3.5: 79 µm Ø 4.0–5.0: 81 µm	89 µm	81 µm	Ø ≤ 3.0: 60 µm Ø ≥ 3.5: 80 µm	81 µm	83 µm	80 µm	Ø ≤ 3.0: 55 µm Ø ≥ 3.5: 65 µm	Ø ≤ 3.0: 84 µm Ø ≥ 3.5: 88 µm
Polymer composition	PLGA	PLGA	Fluorinated Copolymer	Poly-L-lactic Acid	BioLinx™	Polylactic Acid	PDLLA	na	na
Polymer type	Biodegradable	Biodegradable	Durable	Biodegradable	Durable	Biodegradable	Biodegradable	No polymer	No polymer
Polymer thickness	4 µm	4 µm	7.8 μm	3.5 µm (abluminal) 7.4 µm (rest)	5.6 µm	8–11 µm	15 µm	na	na
Polymer degradation delay	4 months	4 months	na	12–24 months	na	6–9 months	3–4 months	na	na
Coating	Abluminal	Abluminal	Circumferential	Circumferential	Circumferential	Abluminal	Abluminal	Abluminal	Abluminal
Stent expansion capacity	Ø 2.25–2.75: 3.5 mm Ø 3.0–3.5: 4.25 mm Ø ≥ 4.0: 5.75 mm	Ø ≥ 3.5: 6.0 mm	Ø ≤ 3.0: 3.75 mm Ø ≥ 3.5: 5.75 mm	Ø ≤ 3.0: 3.5 mm Ø ≥ 3.5: 4.5 mm	Ø 2.25–2.5: 3.5 mm Ø 2.75–3.0: 4.0 mm Ø 3.5–4.0: 5.0 mm Ø 4.5–5.0: 6.0 mm	Ø ≤ 3.0: 3.5 mm Ø ≥ 3.5: 4.5 mm	Ø ≤ 2.5: 3.5 mm Ø 2.75–3.0: 4.5 mm Ø ≥ 3.5: 6.25 mm	Ø ≤ 3.0: 3.5 mm Ø ≥ 3.5: 4.5 mm	Ø ≤ 3.0: 4.76 mm Ø ≥ 3.5: 5.95 mm
Radiopaque Marker position	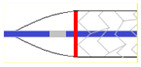	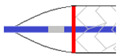	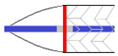	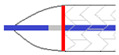	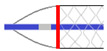	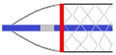	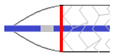	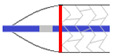	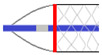

Ø: diameter, PLGA: poly(D,L-lactide-coglycolide), PDLLA: poly(D,L-lactide-co-caprolactone), na: not adapted.
